# Informing the establishment of the WHO Global Observatory on Health Research and Development: a call for papers

**DOI:** 10.1186/1478-4505-13-9

**Published:** 2015-02-02

**Authors:** Taghreed Adam, John-Arne Røttingen, Marie-Paule Kieny

**Affiliations:** World Health Organization, Health Systems and Innovation, 20 Avenue Appia, 1211 Geneva 27, Switzerland; Norwegian Institute of Public Health, Division of Infectious Disease Control, and Institute of Health and Society, University of Oslo, PO Box 4404, N-0403 Oslo, Norway; Harvard T.H. Chan School of Public Health, Boston, USA

**Keywords:** Funding, Global health, Investments, Observatory, Research and development

## Abstract

In May 2013, the WHO Member States requested the WHO to establish a Global Observatory on Health Research and Development (R&D), as part of a strategic work-plan to promote innovation, build capacity, improve access, and mobilize resources to address diseases that disproportionately affect the world’s poorest countries.

The rationale for establishing a Global Observatory on Health R&D is to provide a mechanism to monitor and analyse health R&D resource flows, product pipelines, and research outputs, aiming to contribute to the identification of gaps to inform priority-setting for new R&D investments to be operationalized through a new global financing and coordination mechanism for health R&D and utilized by all stakeholders informing health research policy decisions in countries, civil society, and the private sector.

As one of the mechanisms to achieve the goals of the Global Observatory on Health R&D, the WHO is launching a Call for Papers to be published as a Thematic Series in *Health Research Policy and Systems* to contribute state-of-the-art knowledge and innovative approaches to analyse, interpret, and report on health R&D information. Further, to serve as a key resource to inform the future WHO-convened coordination mechanism, which will be utilized to generate evidence-informed priorities for new R&D investments to be financed through a proposed new global financing and coordination mechanism for health R&D.

**Electronic supplementary material:**

The online version of this article (doi:10.1186/1478-4505-13-9) contains supplementary material, which is available to authorized users.

## Background

After almost a decade since calling for closing the 10/90 gap, a concept put forward by the Commission on Health Research for Development to highlight disparities in research and development support for neglected diseases affecting the world’s poorest countries [1], the World Health Organization (WHO) and its Member States are now united in recognizing the urgency in addressing the health needs of the world’s poorest countries. In particular, the inequities in the current research landscape due to recognized market failures and the need for increasing investments in health research and development (R&D) related to diseases that overwhelmingly or predominantly affect the poor (Type III and Type II diseases), as well as the specific R&D needs of low- and middle-income countries (LMICs) for diseases affecting both high-income countries and LMICs (Type I) [2, 3].

Related to this, in May 2013, the World Health Assembly, in its resolution 22.66, requested the WHO to establish a Global Observatory on Health R&D, as part of a strategic work-plan along with other actions including exploring the possibilities for the development of a global financing and coordination mechanism for health R&D to promote innovation, build capacity, improve access, and mobilize resources to address diseases that disproportionately affect the world’s poorest countries [3]. The ultimate goal is the development and delivery of affordable, effective, safe, and quality health products, especially those for which existing market mechanisms fail to provide incentives for R&D.

The rationale for establishing a Global Observatory on Health R&D is to provide a mechanism to monitor and analyse relevant existing information on health R&D, including resource flows, product pipelines, and research outputs, with a view to contributing to the identification of gaps and opportunities for health R&D and to inform priority-setting for new R&D investments based on the public health needs of the world’s poorest countries.

### Seizing the opportunity for health research and development: introducing a new journal series

To take advantage of this momentum and recognizing the pressing need for high quality evidence on the availability of and gaps in health R&D to inform future allocation of resources [4], the WHO is issuing a Call for Papers inviting interested researchers and institutions to contribute to the knowledge base necessary to inform these decisions, taking advantage of the data made available through the Global Observatory platform and the associated resources on the dedicated WHO website.

The overall goal of this Call is the publication of a selection of papers to contribute to a new peer-reviewed journal Series on “Health R&D”. With such a timely collection of articles and cutting-edge knowledge in this field, the WHO aims to provide global stakeholders with up-to-date knowledge on methods, strategies, tools, experiences, and applications to draw from when developing future investment decisions and implementation plans for new R&D. More importantly, the aim is to push the frontier for knowledge and innovation in this field by inviting new thinking, approaches, analysis, and information, and welcome a wide range of perspectives and disciplines relevant to understanding the availability of and funding for health R&D.

### Scope of the series and the Call for Papers

We welcome primary research in the categories and topics described below. Articles providing new knowledge and innovative techniques will be prioritized as well as analysis of diseases that overwhelmingly or predominantly affect the poor, e.g., neglected diseases. Purely descriptive articles or those that simply seek to argue for the importance of investments on health R&D or make the case for why it matters are not encouraged in the context of this Series. The examples below are to provide some ideas of what would be considered relevant for this Call. Nevertheless, other ideas that fit the purpose and scope of this Call are also welcome.

### Methodologies

This category of papers may include methodological developments, approaches, tools, or explorations to support analysis or understanding of the range of health R&D and related investments, as well as approaches to incorporate the associated learning in the policy making process. This could be a reflection of existing experiences as well as exploration of new ideas.

Some examples of methodological topic areas include:

1. Priority setting for health R&D – methodologies for defining needs (including evidence on burden of disease, evidence on severity of disease, product gaps, demand/market size projections, stakeholder values, etc.)
2. Decision making for funding R&D/methods for selecting new R&D (including technical- and process-related tools, e.g., to determine trade-offs between alternatives, including elements such as market incentives and failures, etc.)
3. Handling financial flow data with a focus on R&D
4. Measuring expenditure data with a focus on R&D
5. Assessing product pipelines (e.g., in terms of quality, sustainability, and relevance to needs and/or priorities)
6. Approaches to assessing research outputs (e.g., bibliometrics or social network analyses)
7. Approaches to assessing research results (e.g., in terms of the range of products and innovation)
8. Forecasting future R&D funding needs
9. Forecasting future R&D funding levels

### Reviews

Reviews of empirical work that discuss or clarify the different approaches to explore the availability, ethics, capacity for, and investments in health R&D. These may include:

- Reviews of ethical considerations in developing new R&D for particular population groups, diseases, or product types
- Resource tracking tool assessments for R&D, for example, for:
- Product pipelines: i) product developers and what they develop; ii) evaluating tracking tools and their strengths and limitations
- R&D funding: i) funders and what they fund; ii) evaluating tracking tools and their strengths and limitations

### Analyses

This category may include analytical work to inform future research or new investment decisions on health R&D, for example:

- Funding trends and R&D gap analyses by disease area/product area/R&D stage
- Landscape analyses of financing mechanisms for incentivizing R&D
- Landscape analyses of platforms for implementing R&D (competitive vs. collaborative models)
- In-depth product development pipeline analyses by disease area/product area/R&D stage
- In-depth product development expenditure analyses by disease area/product area/R&D stage (his could be tailored, for example, by looking at Product Development Partnership expenditures first)
- Bottleneck analyses in R&D (translational, manufacturing, intellectual property, regulatory, etc.)
- Survey-based analyses of stakeholder motivations behind funding and selecting R&D
- Predictive analyses of expected future R&D funding and barriers/success factors to meeting funding needs
- Bibliometric analysis of research outputs, for example, covering:
- What topics, studies, to what extent, by whom
- Research collaborators and their networks

### Utilization

This category may, for example, make use of case studies to share innovative ideas or experiences that have worked in the health R&D field. These may include:

1. Case studies of best-practice models of financing or managing R&D
2. Cases where rigorous monitoring and analyses have been utilized to inform decision making

### How to apply to the call for papers

Interested researchers are invited to submit an abstract to adamt@who.int using the guidelines and template available in Additional file 1 and on the WHO website http://www.who.int/healthsystems/r-d_observatory. The deadline for submission is the 8th of March, 2015 (23:59 GMT). An external scientific committee will select abstracts for potential inclusion in the Series. The final decision for inclusion rests with the editors of *Health Research Policy and Systems*, following the usual peer-review process. The Series aims to cover up to 25 articles of diverse nature and topics. If the desired number and diversity of articles is not reached, the deadline for accepting abstracts for this Call will be extended and will be announced on the WHO website (link above). Selection of additional papers after the deadline will follow the same process.

### Looking forward

There are several needs and opportunities for methodological developments and new information to support analysis and priority setting for health R&D. With this new Thematic Series on health R&D, we hope to encourage researchers and institutions interested in this field to address or advance some of them. The ultimate goal is to inform the establishment of the WHO’s Global Observatory on Health R&D and to guide future decision-making and priority setting in this area.

Global stakeholders have various roles to play to take advantage of the momentum created by the World Health Assembly’s Resolution and the powerful underlying desire to redress the current imbalances in health R&D investments, particularly WHO Member States’ request to the WHO to explore the possibilities for hosting a pooled fund, which would finance priority R&D projects. The priorities of the fund would be informed by the analysis of the research landscape provided by the Global Observatory on Health R&D, including those provided by this Thematic Series, as recommended by the future WHO-convened coordination mechanism [3].

We, therefore, believe that this Series is well positioned to provide timely, high quality, easily accessible, and relevant information to capitalize on this momentum and, more importantly, to serve as a key resource to inform this global agenda with the ultimate goal to address priority research gaps for health R&D that disproportionately affect the world’s poorest countries.

## Electronic supplementary material

Additional file 1: **Guidelines for submission of abstracts.** (DOC 93 KB)

## Abbreviations

LMICs: Low- and middle-income countries
R&D: Research and development
WHO: World Health Organization.
