# Supplementary material for: Informing the establishment of the WHO Global Observatory on Health Research and Development: a call for papers
Source: Health Res Policy Syst. 2015 Feb 2;13:9. doi: 10.1186/1478-4505-13-9 (PMC4328039; doi:10.1186/1478-4505-13-9)
Supplement: Supplementary file 1 — Additional file 1: Guidelines for submission of abstracts. (DOC 93 KB) [file 12961_2015_373_MOESM1_ESM.doc]

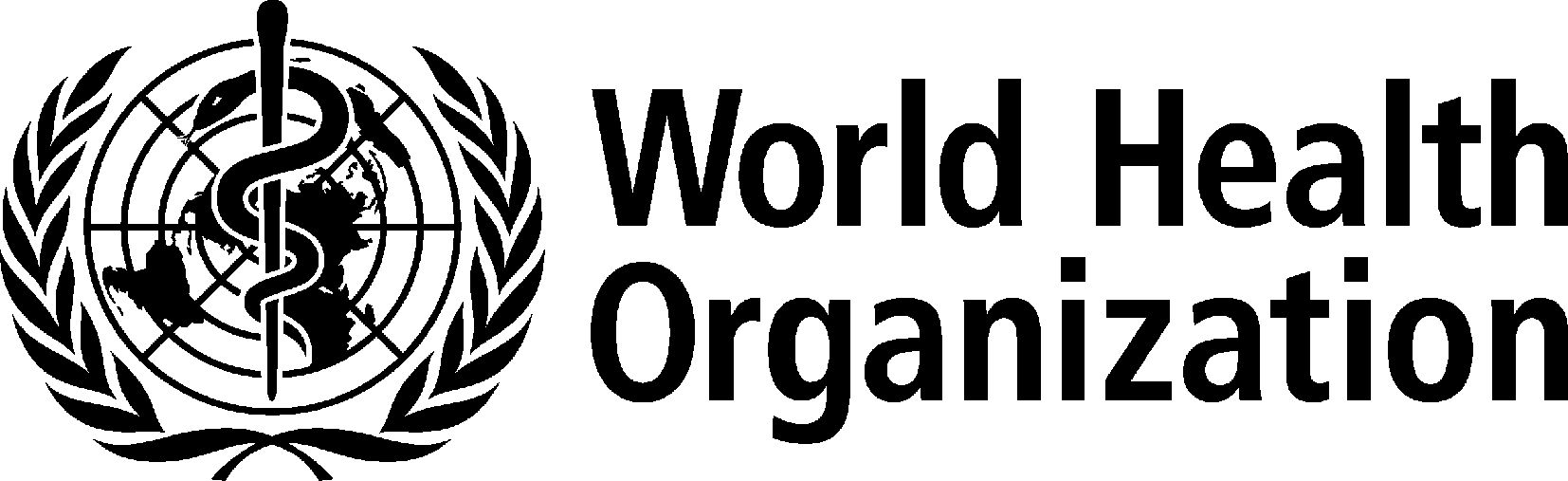


# Call for Papers for a Journal Series on:

# Informing the establishment of the WHO Global Observatory on Health Research and Development

# Guidelines for submission

## Launched in association with:

# The WHO Global Observatory on Health Research and Development

### Deadline for Submission: March 8^th^ , 2015 (23:59 GMT)

# 1. Introduction

# This Call for Papers on “Informing the establishment of the WHO Global Observatory on Health Research and Development” is issued by the Health Systems and Innovation Cluster of the World Health Organization in association with the 2013 WHA resolution 66.22 calling for the establishment of a Global Observatory on Health Research and Development (R&D) to guide future R&D investments. The scope of the Call for Papers are published in an Editorial in *Health Policy Research and systems* (2015): “Informing the Establishment of the WHO Global Observatory on Health Research and Development : A Call for Papers”.

This document outlines the guidelines for submission of abstracts, the criteria for selection and the template that should be used for the submission.

# Guidelines for Submission, Evaluation Process, Criteria for selection and Timelines

Please direct all questions concerning this Call, by email, to Taghreed Adam at [adamt@who.int](mailto:adamt@who.int). In the subject field of the email please use **"Question: Call for Papers on Health R&D”**.

Submissions will be evaluated according to the following criteria:

- Innovation of the proposed topic and research questions, and the value added to existing literature (30%).
- Quality of the abstract, rigour of the proposed research strategies and clarity of arguments and questions being addressed (45%)
- Publication experience of the lead author and writing team (25%).

Where technical criteria are of equal merit, the final decision will be made by taking into account the diversity of topics, research institutions and research teams from LMICs.

All submissions must be sent by email ([adamt@who.int](mailto:adamt@who.int)), and must be received by the **8^th^ of March, 2015 (23:59 GMT).** Submissions should be in the format provided in Annex 1. In the subject field of the email please use **"Application: Call for Papers on Health R&D”**. WHO will notify all applicants of receipt of their application. Please contact us if you have not received a notification within a week of your submission.

Submissions will be independently reviewed by two technical experts using the criteria described above. A small external scientific committee will make the final selection.

Successful applicants will be **notified by mid April 2015.** WHO will inform the Journal of the selected abstracts and teams for potential inclusion in the Thematic Series (upon successful peer-review). Consequently, research teams will be encouraged to submit their articles to the *Health Policy Research and Systems* as soon as possible to undergo the peer-review process. Instruction for submitting to the Journal will be communicated to the successful teams. Articles accepted for publication will be published as soon as ready. It is expected that the publication of the Series of papers will span from the second half of 2015 till the end of 2016.

# Submission Format

Submissions must be sent in English. Please see Annex 1 for the application format (also available in a word format on the website) and for guidance on what information should be included.

# Annex 1 - Application Template

THIS FORM SHOULD BE SUBMITTED BY E-MAIL TO: [adamt@who.int](mailto:adamt@who.int)

| **Selected information from this box (1.1-1.3) may be released to the general public if this paper is selected** | | | |
| --- | --- | --- | --- |
| **1.1 Name of lead author and institutional affiliation**: | | | |
| Title: | Surname: | | First name: |
| Name of Department and Institution (30 words maximum) | | | |
| Full postal address of team leader to be used for correspondence (170 words maximum): | | | |
| Telephone (Fixed): | | Telephone (mobile): | |
| E-mail: | | E-mail 2: | |
| **1.2 Title of manuscript:**  ID Number:     [LEAVE BLANK] | | | |
| **1.3 Abstract:** ( 1000 words maximum). *See guidance on what information to include in the footnote at the end of this form^1^.* | | | |
| **1.4 Full names, affiliations and field of expertise of co-authors (knowing that this can change as the manuscript develops)**  a.  b.  c.  d.  e. | | | |
| **1.5 List of main publications**  Please provide the full reference of publications by the lead author and one (or two) of the main co-authors, preferably the most relevant to this Call (up to five references will be sufficient).  a.  b.  c.  d.  e. | | | |

^1^ There is no specific format for the abstract section, but the word count should not exceed 1000 words. This section is critical to the selection process so please focus on information that clarifies the research question/s you intend to explore in this manuscript, and how you intend to address them. As relevant, briefly outline the overarching research approach, what methods or tools will be used to address your research questions, the anticipated outcome and new knowledge provided through this manuscript. You can also use this space to briefly explain why the research is relevant to future research or policy making in this area.
